# THRIVE‐c score predicts clinical outcomes in Chinese patients after thrombolysis

**DOI:** 10.1002/brb3.927

**Published:** 2018-01-30

**Authors:** Yuesong Pan, Yujing Peng, Weiqi Chen, Yongjun Wang, Yi Lin, Yan He, Ning Wang, Yilong Wang

**Affiliations:** ^1^ Department of Epidemiology and Health Statistics School of Public Health Capital Medical University Beijing China; ^2^ Beijing Municipal Key Laboratory of Clinical Epidemiology Beijing China; ^3^ Department of Neurology Beijing Tiantan Hospital Capital Medical University Beijing China; ^4^ China National Clinical Research Center for Neurological Diseases Beijing China; ^5^ Center of Stroke Beijing Institute for Brain Disorders Beijing China; ^6^ Beijing Key Laboratory of Translational Medicine for Cerebrovascular Disease Beijing China; ^7^ Department of Neurology and Institute of Neurology The First Affiliated Hospital of Fujian Medical University Fuzhou China

**Keywords:** ischemic, prognosis, stroke, THRIVE‐c score, thrombolysis

## Abstract

**Objectives:**

Total Health Risks in Vascular Events‐calculation score (THRIVE‐c) is an easy use and patient‐specific outcome predictive score by computing the logistic equation with patients’ continuous variables. We validated its performance in Chinese ischemic stroke patients receiving intravenous thrombolysis (IVT) therapy.

**Materials and Methods:**

We used data from the Thrombolysis Implementation and Monitor of Acute Ischemic Stroke in China (TIMS‐China) registry to validate the THRIVE‐c score in patients receiving IVT therapy. We evaluated the score performance using area under the receiver operating characteristic curve (AUC). Receiver operator characteristic curve (ROC) was used to compare THRIVE‐c score performance with other scores in predicting clinical outcome and symptomatic intracranial hemorrhage (SICH). Calibration was assessed by Pearson correlation coefficient and Hosmer–Lemeshow test.

**Results:**

Among the 1,128 patients receiving IVT therapy included in this study, AUC of the THRIVE‐c score for 3‐month SICH, poor functional outcome, and mortality rate was 0.70 (95% CI: 0.63–0.76), 0.75 (95% CI: 0.73–0.78) and 0.81 (95% CI: 0.77–0.85), respectively. The increased THRIVE‐c score was associated with higher risk of developing SICH, poor functional outcome, or mortality in patients with acute ischemic stroke at 3 months after thrombolysis. The performance of the THRIVE‐c score was similar to or superior to other predictive scores (THRIVE score, SEDAN score, DRAGON score, HIAT2 score).

**Conclusions:**

The THRIVE‐c score reliably predicts the risks of 3‐month SICH, poor functional outcome, and mortality after IVT therapy in Chinese patients with ischemic stroke.

## 1 INTRODUCTION

Intravenous thrombolysis (IVT) using recombinant tissue plasminogen activator (rt‐PA, alteplase) is one of the most effective therapy for patients with acute ischemic stroke (AIS) within 4.5 hr (Bluhmki et al., 2009; Hacke et al., 2008; Huang et al., 2015). However, symptomatic intracranial hemorrhage (SICH) is the big concern of using rt‐PA (Derex & Nighoghossian, 2008), and it was reported that 5.8% patients developed SICH due to the thrombolysis therapy (Wardlaw et al., 2012; Yaghi et al., 2015). SICH may limit the implementation of the effective treatment with rt‐PA for physicians. On the other hand, the occurrence of SICH may dramatically increase the mortality rate and poor functional outcome (Mazya et al., 2012; Wahlgren et al., 2007). A predicting tool for assessing clinical benefit and SICH risk after intravenous thrombolytic treatment might be helpful (Whiteley et al., 2014).

The previous study reported that Asian patients with standard‐dose rt‐PA had a high risk of SICH, which was different from that in western population (Anderson et al., 2016; Chao et al., 2010; Menon et al., 2012). The Total Health Risks in Vascular Events‐calculation (THRIVE‐c) score was initially developed and validated to predict the risks of developing SICH in western patients receiving intravenous rt‐PA treatment (Flint et al., 2015). It was an easy‐to‐use prediction score, which could help physicians to evaluate the risk of patients developing SICH before intravenous rt‐PA therapy. However, it has not been validated in nonwestern populations.

The aim of this study was to examine the performance of the THRIVE‐c score in Chinese AIS patients receiving intravenous rt‐PA treatment to predict risks of 3‐month SICH, poor functional outcome, and mortality.

## 2 MATERIALS AND METHODS

### 2.1 Data source and subjects

Data of our analyses were derived from the Thrombolysis Implementation and Monitor of Acute Ischemic Stroke in China (TIMS‐China) study. TIMS‐China was a nationwide prospective stroke registry study of consecutive patients who received rt‐PA treatment admitted to 67 hospitals within 4.5 hr after the onset of symptoms. The trial design was described in detail before (Liao et al., 2013). We included patients applying the following inclusion and exclusion criteria: (i) were between 18 and 80 years of age; (ii) received a clinical diagnosis of stroke; (iii) had a cerebral tomographic (CT) or magnetic resonance imaging (MRI) scan ruled out hemorrhage, major ischemic infarction, or other nonischemic diseases; and (iv) had no contraindication for thrombolysis therapy. We collected information on demographic data, clinical data from TIMS‐China. Physicians were trained with standard case report form after obtaining informed consent for participating in the registry and thrombolysis treatment. The follow‐up duration was 3 months, and the information was collected through face to face or telephone. The TIMS‐China was approved by the Ethics Committee of Beijing Tiantan Hospital.

### 2.2 Clinical outcomes

The clinical outcomes included risks of SICH, functional outcome, and mortality at 3 months after thrombolysis. The definition of SICH was a hemorrhage that was not seen on a previous CT scan, and there had subsequently been either a suspicion of hemorrhage or any decline in neurologic status, according to the criteria of the National Institute of Neurological Disorders and Stroke (NINDS) recombinant tissue‐type plasminogen activator stroke study (Kwiatkowski et al., 1995). Poor functional outcome was defined as a modified Rankin Scale score (mRS) of 3–6 (Flint et al., 2013; Kamel et al., 2013), while good functional outcome was defined as a mRS of 0–2 (Flint et al., 2013, 2014). Mortality included death from all causes. All clinical outcomes were determined by at least two neurologists based on neuroimaging and clinical feature. When there was a disagreement, a third senior neurologist would be consulted to reach a consensus decision.

### 2.3 THRIVE‐c score and other predictive scores

The THRIVE‐c score was a multivariable logistic regression models constructed by entering continuous age, continuous NIHSS and dummy variables with natural coding for Chronic Disease Scale (CDS) (the presence or absence of HTN, DM, or AF) levels of 1, 2, and 3. NIHSS score was assessed by neurologists when the patients arrived at emergency room. Hypertension was defined as current history of hypertension, oral antihypertension drugs, or systolic blood pressure ≥140 mm Hg and diastolic blood pressure ≥90 mm Hg (Lei et al., 2014). Diabetes mellitus was defined as a history of diabetes mellitus, with or without the use of antidiabetic medication. Atrial fibrillation was defined as a history of persistent or paroxysmal atrial fibrillation or confirmed by at least one electrocardiogram or the presence of atrial fibrillation during hospitalization (Lei et al., 2014). Other clinical predictive scores were calculated as follows.

The traditional Total Health Risks in Vascular Events (THRIVE) score was calculated from age, initial stroke severity on the NIHSS score, and CDS. The THRIVE score assigned 1 point for age 60–79 years, 2 points for age ≥80 years, 2 points for NIHSS score 11–20, 4 points for NIHSS score ≥21, and 1 point for each CDS component (Flint, Cullen, Faigeles, & Rao, 2010).

Sugar, Early infarct signs, Dense artery, Age, NIHSS (SEDAN) score assigned 1 point for baseline glucose 8.1–12.0 mmol/L, 2 points for glucose >12.0 mmol/L, 1 point for early infarct signs, 1 point for (hyper) dense cerebral artery sign on admission computed tomography scan, 1 point for age >75 years, and 1 point for NIHSS score ≥10 at admission (Strbian et al., 2012).

Dense artery, rankin score, Age, Glucose, Onset to treatment time, NIHSS (DRAGON) score also ranged 0–10 points and assigned 1 point to each of (hyper) dense cerebral artery sign or early infarct signs on admission CT scan, 1 point for prestroke mRS score >1, 2 points for age ≥80 years, 1 point for 65–79, 1 point for baseline glucose level >8 mmol/L, 1 point for onset to treatment time >90 min, and 3 points for baseline NIHSS score >15, 2 points for NIHSS 10–15, 1 point for NIHSS is 5–9 (Strbian et al., 2012).

Houston Intra‐Arterial Therapy 2 score (HIAT2) ranged 0–10 points and assigned 2 points for age 60–79 years, 4 points for age ≥80 years, 1 point for glucose ≥8.3 mmol/L, 1 point for NIHSS score 11–20, 2 points for NIHSS score ≥21, and 3 points for Alberta Stroke Program Early CT Score ≤7 (Sarraj et al., 2013).

### 2.4 Statistical analysis

The continuous and categorical variables of patients’ baseline characteristics in TIMS‐China and original cohort were presented as mean ± *SD* or median (interquartile range, IQR) and percentages, respectively. Baseline variables between patients included in TIMS‐China and original cohort were compared with chi‐squared test for categorical variables. The normality of all continuous variables was tested with the Shapiro–Wilk test. Odds ratios (ORs) with its 95% confidence intervals (CIs) were calculated using multivariable logistic regression. The probabilities of THRIVE‐c were calculated using the logistic equation (Flint et al., 2015). We tested the performance of the THRIVE‐c score by estimating their discrimination and calibration. The discriminatory power of the THRIVE‐c score was assessed by the AUCs and 95% CIs. An AUC statistic of 1.0 indicated perfect prediction, and of 0.5 indicated no better than random prediction. The Z test was used to compare the AUCs of different scores including the THRIVE‐c score, the traditional THRIVE score, and other predictive scores. Calibration was assessed using Pearson correlation coefficient and Hosmer–Lemeshow test. The α level of significance was *p* < .05 two sides. All analyses were performed with SAS software version 9.3 (SAS Institute Inc, Cary, NC, USA).

## 3 RESULTS

### 3.1 Patient characteristics

Among 6,194 patients in original cohort, a total of 1,128 patients were enrolled in TIMS‐China registry from 67 centers in China between May 2007 and April 2012. The baseline characteristics of patients included in TIMS‐China and original cohort were not well balanced. Detail data were summarized in Table 1, which displayed patient age, NIHSS, CDS, and clinical predictive scores (THRIVE‐c score, THRIVE score, SEDAN score, DRAGON score, and HIAT2 score). The patients enrolled had a slightly lower proportion of women and history of diabetes, hypertension, and atrial fibrillation.

**Table 1:** Characteristics of patients in TIMS‐China and original cohort that developed the THRIVE‐c score

Characteristics	TIMS‐China (*n* = 1,128)	Original cohort (*n* = 6,194)	*p*
Female, *n* (%)	440 (39.01)	5,183 (42.5%)	<.001
Age, years, median (IQR)	64 (56–73)	70 (60–76)	–
NIHSS, median (IQR)	11 (7–16)	12 (8–17)	–
CDS	1 (0–1)	1 (0–2)	–
Hypertension, *n* (%)	667 (59.13)	4,042 (66.1%)	<.001
Diabetes Mellitus, *n* (%)	196 (17.38)	1,187 (19.3%)	.16
Atrial Fibrillation, *n* (%)	202 (17.91)	1,504 (24.6%)	<.001
Poor functional outcome^a^ (3 months)	462 (41.81)	2,944 (50.3%)	<.001
Mortality (3 months)	115 (10.39)	–	
SICH (3 months)	61 (5.41)	–	
THRIVE‐c, median (IQR)	0.6148 (0.3492–0.7824)	–	
THRIVE, median (IQR)	3 (2–4)	3 (2–5)	–
SEDAN, median (IQR)	1 (1–2)	–	
DRAGON, median (IQR)	4 (3–5)	–	
HIAT2, median (IQR)	2 (1–3)	–	

CDS, Chronic Disease Scale, 1 point each for presence of presence of HTN, DM, or AF; DRAGON, Dense Artery, Rankin Score, Age, Glucose, Onset to Treatment Time, HIAT2, Houston Intra‐Arterial Therapy 2 score; IQR, interquartile range; NIHSS; NIHSS indicates National Institutes of Health Stroke Scale; SEDAN, Sugar, Early Infarct Signs, Dense Artery, Age, NIH Stroke Score; SICH, symptomatic intracranial hemorrhage; THRIVE‐c, Totaled Health Risks in Vascular Events‐calculation; THRIVE, Totaled Health Risks in Vascular Events.

a Poor functional outcome was defined as a modified Rankin Scale score of 3–6.

### 3.2 THRIVE‐c score and risk of SICH, poor functional outcome, and mortality

The THRIVE‐c model was a logistic equation using fixity coefficients showed as follows

$$P=\frac{1}{1+e^{-\left(4.94+\left(-0.035*\mathrm{age}\right)+\left(-0.19*\mathrm{NIHSS}\right)+\left(-0.11*\mathrm{CDS}1\right)+\left(-0.41*\mathrm{CDS}2\right)+\left(-0.70*\mathrm{CDS}3\right)\right)}}$$

where age and NIHSS were integer values, and CDS1, CDS2, and CDS3 were virtual variables encoding the state of the CDS for a given subject (Flint et al., 2015).

**Figure 1:**
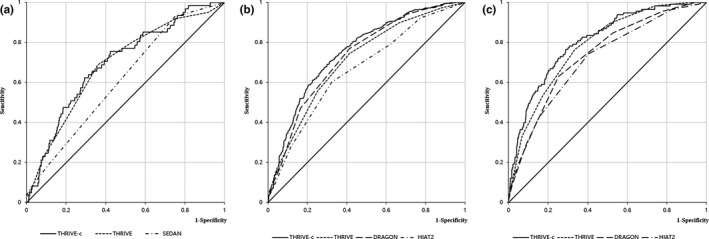
Receiver operator characteristic (ROC) curve analysis comparing THRIVE‐c score with other predictive scores. ROC curves for prediction of SICH (a), poor functional outcome (b), and death at 3 months. DRAGON, Dense Artery, Rankin Score, Age, Glucose, Onset to Treatment Time, HIAT2, Houston Intra‐Arterial Therapy 2 score NIHSS; SEDAN, Sugar, Early Infarct Signs, Dense Artery, Age, NIH Stroke Score; THRIVE‐c, Totaled Health Risks in Vascular Events‐calculation; THRIVE, Totaled Health Risks in Vascular Events

As THRIVE‐c score increasing, the risk of 3 months SICH after thrombolysis increased (Figure 1a). Rates of poor functional outcome (defined as mRS 3–6) and mortality after thrombolysis therapy at 3 months were also on the rise depending on the increasing of THRIVE‐c score (Figure 1b,c). Logistic regression showed that patients with higher THRIVE‐c score were associated with higher rates of both poor functional outcome and death which were similar to SICH (Figure 1).

### 3.3 Receiver operator characteristic curve analysis comparing THRIVE‐c score with other predictive scores

The AUC of receiver operator characteristic (ROC) curve for THRIVE‐c score was similar to the THRIVE score in predicting SICH, while it was superior to the SEDAN score (Figure 1a). For predictive ability of thrombolytic SICH, the AUC of THRIVE‐c score was 0.70, compared with 0.69 for THRIVE score (*p* = .63), 0.61 for SEDAN score (*p* = .0032) (Table 2).

**Table 2:** Comparison of AUCs among THRIVE‐c score and other predictive scores for symptomatic intracranial hemorrhage, poor functional outcome and mortality at 3 months

Outcome	AUC (95% CI)	*p* Value
SICH (3 months)		
THRIVE‐c	0.70 (0.63–0.76)	
THRIVE	0.69 (0.62–0.75)	.63
SEDAN	0.61 (0.55–0.68)	.0032
Poor functional outcome^a^ (3 months)		
THRIVE‐c	0.75 (0.72–0.78)	
THRIVE	0.71 (0.68–0.74)	<.0001
DRAGON	0.73 (0.70–0.76)	.072
HIAT2	0.66 (0.63–0.70)	<.0001
Mortality (3 months)		
THRIVE‐c	0.81 (0.77–0.85)	
THRIVE	0.78 (0.74–0.82)	.039
DRAGON	0.74 (0.69–0.78)	<.0001
HIAT2	0.71 (0.66–0.76)	<.0001

AUCs, Area Under the Receiver Operator Curves; CI, Confidence Interval; DRAGON, Dense Artery, Rankin Score, Age, Glucose, Onset to Treatment Time, NIHSS; HIAT2, Houston Intra‐Arterial Therapy 2 score; SEDAN, Sugar, Early Infarct Signs, Dense Artery, Age, NIH Stroke Score; SICH, symptomatic intracranial hemorrhage; THRIVE‐c, Totaled Health Risks in Vascular Events‐calculation; THRIVE, Totaled Health Risks in Vascular Events.

a Poor functional outcome was defined as modified Rankin Scale 3–6 at 3 months.

The AUC of ROC curve for THRIVE‐c score was similar to the DRAGON score but greater than other outcome predictive scores (THRIVE, HIAT‐2) in predicting poor functional outcome (Figure 1b). For poor functional outcome prediction, the AUC of THRIVE‐c score was 0.75, compared with 0.71 for THRIVE (*p* < .0001), 0.73 for DRAGON (*p* = .072), 0.66 for HIAT2 (*p* < .0001).

Moreover, the AUC of ROC curve for THRIVE‐c score had an advantage over other outcome predictive scores (THRIVE score, DRAGON score, and HIAT2 score) in predicting death at 3 months (Figure 1c). For mortality prediction, the AUC of THRIVE‐c score was 0.81, compared with 0.78 for THRIVE score (*p* = .039), 0.74 for DRAGON score (*p* < .0001), 0.71 for HIAT2 score (*p* < .0001).

### 3.4 Calibration ability of THRIVE‐c score

**Figure 2:**
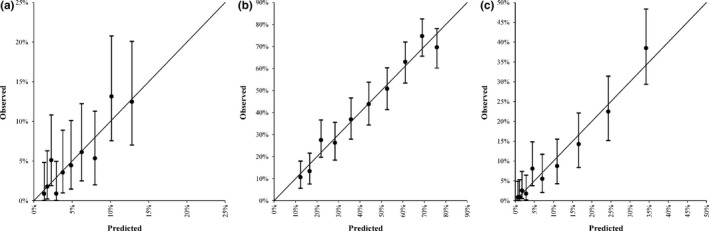
Calibration plot of THRIVE‐c score for (a) symptomatic intracranial hemorrhage, (b) poor functional outcome, and (c) death at 3 months. The vertical lines indicate the 95% confidence intervals of predicted rates of clinical outcome

Calibration analysis of THRIVE‐c score showed a high correlation between predicted and observed probability of SICH (*r* = .91, *p* < .001), 3‐month poor functional outcome (*r* = .98, *p* < .001), and mortality (*r* = .98, *p* < .001). The significance level of the Hosmer–Lemeshow test for the prediction of SICH was 0.77 (Figure 2a). And for the prediction of poor functional outcome and death, the significance level of the Hosmer–Lemeshow test was 0.14 and 0.56, respectively (Figure 2b,c).

## 4 DISCUSSION

Our study showed that the THRIVE‐c score strongly predicted risks of developing SICH, poor functional outcome, and mortality among AIS patients after receiving thrombolysis in Chinese population. The performance of the THRIVE‐c was similar to or superior to other predictive scores (THRIVE score, SEDAE score, DRAGON score, and HIAT‐2 score).

Furthermore, our study showed that the THRIVE‐c score was better in predicting mortality than developing SICH at 3 months. The possible explanation was that the patients’ profiles were rather complete in predicting poor functional outcomes and mortality, whereas some crucial risk factors including pretreatment blood pressure, usage of antiplatelets, and statins were absent in predicting SICH.

Compared with the traditional clinical scoring systems, the great advantage of the THRIVE‐c score was improvement in accuracy, using continuous predictors instead of predictors that had been cut to generate a simplified scoring system. Secondly, the greater degree of granularity derived from model‐estimated outcome probability may also have advantages in clinical trial. For example, instead of defining cut points for continuous variables like age as inclusion criteria for a clinical trial, researchers could define a “prerandomization probability” of good outcome (based on the appropriate calculation) as a threshold for inclusion. Thirdly, directly mode‐estimated outcome maybe provide better interoperability between different prediction systems. For example, THRIVE‐c calculated directly prior to acute stroke intervention could serve as a “preintervention probability” in Bayesian analysis in which a likelihood ratio as regards to intervention data (such as time to recanalization, recanalization, and/or extent of collaterals) could be used to calculate a postintervention probability of good outcome for a given patient (Jaeschke, Guyatt, & Sackett, 1994).

The THRIVE‐c score is an easy‐to‐use tool based on the patient's medical history and physical examination. Compared with the SEDAN score, the THRIVE‐c score did not require serum glucose levels for predicting post‐thrombolysis SICH (Strbian et al., 2012). And compared with the DRAGON and HIAT2 score, the THRIVE‐c score did not require interpretation of neuroimaging findings such as the Alberta Stroke Program Early CT (ASPECTS) sore in HIAT2 (Sarraj et al., 2013) and the hyperdense artery sign and early infarct signs in DRAGON (Strbian et al., 2012).

Our study had several limitations. First, most of the participating hospitals in TIMS‐China were urban hospitals with more resources and experts than hospitals in rural areas. Thus, the study could contain selection bias. Second, changes in medical services during the 5‐year study period might have influenced the study results. Third, the THRIVE‐c score did not include information on imaging and laboratory results, which might affect the prognosis of patients. Fourth, the mortality in our study was included death from all causes which was not limited to that caused by stroke. Finally, the AUC of the THRIVE‐c score in our study did not reach the threshold of 0.8, which was required for using on individuals. But the prognostic outcome of our study was very close to 0.8 which was still relatively reliable. Thus, we felt confident of its use in clinical practice.

Our study showed that the THRIVE‐c score was a reliable and accurate tool for clinicians to predict risks of SICH, poor functional outcome, and mortality after thrombolysis therapy in Chinese acute ischemic stroke patients.

## CONFLICT OF INTEREST

The authors declare that they have no competing interests.
